# Assessing the impact of history of medicine research: A scientometric and altmetric analysis

**DOI:** 10.1002/hsr2.2186

**Published:** 2024-07-01

**Authors:** Jamal Rezaei Orimi, Mohammad Hossein Asadi, Forouhe Jafari, Aboozar Ramezani, Seyyed Amir Hossein Latifi, Azam khosravi, Seyed Abdollah Mahmoodi, Mehdi Salehi, Hasan Siamian

**Affiliations:** ^1^ Traditional and Complementary Medicine Research Center Arak University of Medical Sciences Arak Iran; ^2^ Department of History of Medical Sciences Arak University of Medical Sciences Arak Iran; ^3^ Department of History of Medical Sciences School of Persian Medicine, Qom University of Medical Sciences Qom Iran; ^4^ Medical librarian and Information Sciences, Department of Scientific Publications and Information Development Center (SPIDC), Vice Chancellery for Research & Technology Iran Ministry of Health and Medical Education Tehran Iran; ^5^ Department of Islamic Studies, School of Medicine Arak University of Medical Silences Arak Iran; ^6^ Department of Traditional Medicine Traditional and Complementary Medicine Research Center (TCMRC), School of Medicine Arak Iran; ^7^ Department of Health Information Technology, School of Allied of Medical Sciences, Mazanadaran University of Medical Sciences Sari Iran

**Keywords:** bibliometrics, cluster analysis, history of medicine, PubMed

## Abstract

**Background and Aims:**

After conducting a comprehensive literature search of two medical electronic databases, PubMed and Embase, as well as two citation databases, Web of Science Core Collections (WoS) and Scopus, we aimed to conduct an Altmetric and Scientometric analysis of the History of Medicine literature in medical research.

**Methods:**

The following software tools were used for analyzing the retrieved records from PubMed and Embase databases and conducting a collaboration analysis to identify the countries involved in scientific medical papers, as well as clustering keywords to reveal the trend of History of Medicine research for the future. These software tools (VOSviewer 1.6.18 and Spss 16) allowed the researchers to visualize bibliometric networks, perform statistical analysis, and identify patterns and trends in the data.

**Results:**

Our analysis revealed 53,771 records from PubMed and 54,405 records from EMBASE databases retrieved in the field of History of Medicine by 105,286 contributed authors in WoS. We identified 157 countries that collaborated on scientific medical papers. By clustering 59,995 keywords, we were able to reveal the trend of History of Medicine research for the future. Our findings showed a positive association between traditional bibliometrics and social media metrics such as the Altmetric Attention Score in the History of Medicine literature (*p* < 0.05).

**Conclusion:**

Sharing research findings of articles in social scientific networks will increase the visibility of scientific works in History of Medicine research, which is one of the most important factors influencing the citation of articles. Additionally, our overview of the literature in the medical field allowed us to identify and examine gaps in the History of Medicine research.

## 1 INTRODUCTION

One of the requirements for effective and correct policy making in medical research is to identify the current situation and the strengths and weaknesses of scientific movements. Hence, Scientometric indicators can be an essential tool to provide information to planners and policymakers. The relationship between journals, articles, and authors is often investigated with traditional scientometric indicators, such as direct citation, co‐citation, and bibliographic coupling, which are three scales to measure the relationship between research entities.^1^, ^2^ For example, the number of citations indicates the approval, impact, quality, or reputation of the article and the authors. Nowadays, Altmetric indicators are also used to evaluate or investigate the research impact of the fields.^3^

The measurement and facilitation of social media impact are discussed in the Almetrics literature.^3^ In other words, the media or social networks are among the web resources recently used as an auxiliary tool for the publication, dissemination, and evaluation of research activities that have attracted increasing attention and seem can present a more comprehensive picture of scientific influence. For example, the number of times a scientific paper's findings have been cited in nonscientific contexts, bookmarked, liked, or downloaded, whether in social media, policy documents, popular encyclopedia articles, news outlets, or blogs.^4^, ^5^

There are many tools in Altmetric studies to measure the effectiveness of research outputs, such as the Altmetric Explorer, ImpactStory, Mendeley, ResearchGate, CrossRef, and PlumX. Providers of altmetrics may list these interactions, or they may offer a composite index calculating a single altmetric score attributed to the publication.^3^, ^6^ For example, Altmetric company limited liability partnership is a start‐up business from London, UK, developing tools to gather online attention surrounding scholarly content from many sources. (Note: it is in this general sense that the term “altmetrics” is used in this paper; references to the popular implementation called Altmetric will be written with a capitalized “A”).

Since the history of medicine as a research field plays an essential and critical role in future‐oriented research, it should not be neglected to conduct related research.^7^ To maintain a proper balance between the latest knowledge and practice, it is necessary to navigate medical history research in the correct direction and use its results ideally.^8^ A significant part of medical history research is done by faculty members and professors related to medical history in the form of scientific articles. Many of the scientific productions carried out by faculty members, professors, and medical history researchers at different levels of education are research that have been carried out scientifically. Since it is possible to develop medical history and change its policies based on research findings, it is necessary to select research topics based on priorities and needs related to the health of the society so that the spent resources will have an effective result.^7^

In this regard, medical history research is an effort to recognize the human medical heritage and its evolution, the contribution of nations to the innovations in past medical knowledge and its effects on today's medical sciences, and to analyze and find the root causes of its progress and decline. The use of research results in today's medical history is a professional responsibility closely related to the concept of quality improvement. Therefore, the researchers of the history of medical sciences targeted interventions should be made to incline the benefits of using the research results. In addition, the correct use of research‐based evidence and findings, in addition to improving the quality and credibility of medical history research, makes medical history researchers accountable for their performance.^7^, ^8^, ^9^ Therefore, since there has been no comprehensive research on the quantity of scientific production of researchers related to the history of medicine, we are facing many uncertainties and research gaps in this regard. Our study determines the number of scientific productions about the history of medicine, the status of countries, and the position of researchers, universities, and institutions.

While several bibliometrics studies were conducted related to the History in general, to our knowledge, no scientometric investigation focused on the result related to the History of Medicine. This study performs bibliometrics of the History of Medicine in the medical database to cover this gap. PubMed and EMBASE are used to visualize scientific productions in the field. The secondary purpose was to evaluate the correlation between Altmetric studies and traditional citation‐based metrics. The Research questions:

1. What is the visualization of scientific productions in the field of History of Medicine in databases?
2. What is the presence of research articles in the field of History of Medicine on social media? (Is there a significant positive correlation between citations rate of articles and presenting in social media?)

## 2 METHODS

To fulfill the primary purpose, descriptive data is presented. To identify or choose medical keywords and databases, general planning has been developed. The search query process is initiated by identifying the “history of medicine” keyword. For instance, “History of Medicine”[Majr] is identified by MeSH term thesaurus restricted to major topics, and PubMed will automatically search synonyms and other entry terms such as “Medicine Histories” and “History Medicines” (Supp1: MeSH & Emtree). Descriptive statistics can be useful for two purposes: 1) to provide basic information about variables in a data set and 2) to highlight potential relationships between variables.

A comprehensive literature search will be conducted of two electronic databases: PubMed (1900–2021), Embase (1900–2021). Also, we selected two citation databases to conduct the research: Web of Science Core Collections (WoS) and Scopus. These databases are the most comprehensive research sources in medicine and contain high‐quality peer‐reviewed journals.^10^ The statistical population of this research is all the scientific productions indexed in the medical databases, including PubMed and EMBASE, in the field of “history of medicine” without a time limit. The Language was no limited for PubMed and EMBASE in the search options. Data range time was expanded all years to Jul 2022. Search strategies in PubMed and EMBASE used thesaurus terms. The document type in PubMed and EMBASE was unlimited. Table 1 shows the search options for PubMed and Embase.

**Table 1:** The search options for PubMed and Embase.

Database	Criteria	Results
PubMed	Data range: all years until 2022	53,595
	Language: no limited	
	Query search:	
	Mesh terms search in PubMed	
	All document type	
Embase	Data range: all years until 2022	54,111
	Language: no limited	
	Query search: history of medicine'/exp OR ‘history of medicine’/mj	
	All document types	

**Figure 1:**
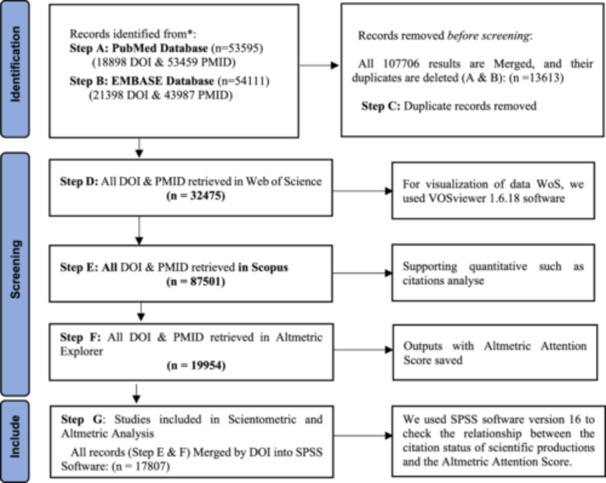
The flow diagram of the different steps of data collection and analysis.

Steps A & B in Figure 1 illustrates the flow diagram of the different steps undertaken for this process.^11^ In other words, another step has been carried out parallel to the first. From the total of articles in two medical databases, of 107,706 records, 13,613 duplicate records identified by EndNote software.

The first steps imported 107,706 articles in the endnote software from two databases, and we removed 13,613 duplicated records. Then, the DOI and PMID of remained articles were used to retrieve citation information (Figure 1 Steps A, B and C).

We did step D to illustrate the visualization. Web of Science Core Collections (WoS) includes Science Citation Index Expanded (SCI‐EXPANDED)‐1970‐present, Social Sciences Citation Index (SSCI)‐1970‐present, Arts & Humanities Citation Index (AHCI)‐1975‐present, Emerging Sources Citation Index (ESCI)‐2017‐present. We only had access to the data of WoS in the time range of 1975 until 2022. So, by a bibliometric method, the first research purpose of this study has been answered. We visualized the most influential researchers and countries. Furthermore, the core research subject was revealed by clustering keyword co‐occurrence. For visualization of data WoS, we used VOSviewer 1.6.18 software (Figure 1 step D).

Step E involved finding citation information from Elsevier's Scopus database, a comprehensive global and regional bibliometric data source for scientific journals and conference proceedings, supporting quantitative science investigations. Then, for step F, social media mentions extracted from Altmetric Explorer.* Total mentions in some social media were divided into five categories by Altmetric Explorer: social media (Twitter, Facebook, Google+, Reddit posts, Sina Weibo, Pinterest, LinkedIn), other sources(Wikipedia, Videos, Q&A posts), news and blogs(News, Blogs), policy and patents(Patents, Policy documents), and academic sources(Faculty Opinions, Peer reviews) (Figure 1F). At first, DOI and PMID were extracted from all records in PubMed & EMBASE databases; then, we explored data from the Altmetric Explorer for all research outputs. We retrieved Altmetric data on 25 September 2022. (Figure 1 Step E & F).

After saving the mentioned records of Altmetric Explorer, according to the second research purpose, we used SPSS software version 16 to check the relationship between the citation status of scientific productions and the Altmetric Attention Score. Therefore, we used Spearman's non‐parametric statistic test for the second question. The *p* < 0.05 was considered statistically significant. The Spearman correlation coefficient (r) <0.3 was interpreted as poor, 0.3–05 as low, 0.5–0.7 as moderate, 0.7–0.9 as high, and >0.9 as very high.^12^ (Figure 1 Step G).

## 3 RESULTS

### 3.1 Medical databases

**Figure 2:**
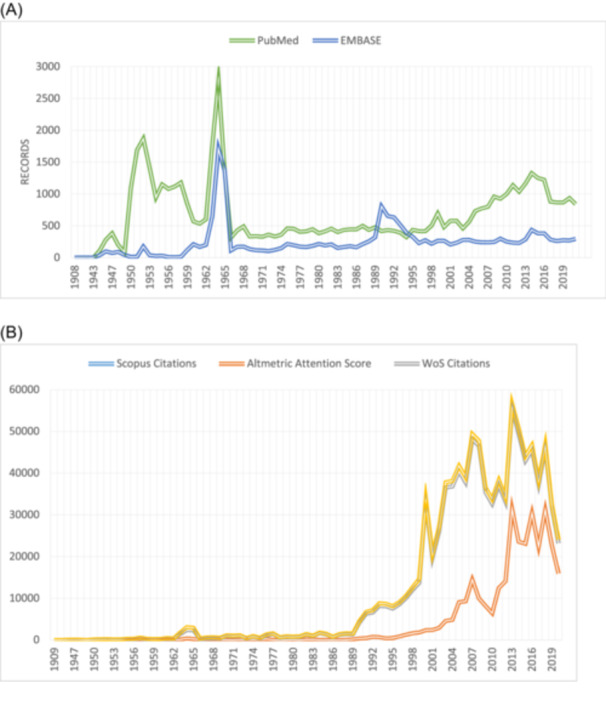
Status of History of Medicine articles in different databases until 16 August 2022. (A) Annual publications' distribution History of Medicine articles based on PubMed and Embase databases. (B) Impact of research with Scopus and Altmetric Explorer databases.

Figure 2A shows the 53771 records of PubMed and 54405 records of EMBASE databases. The number of publications has variable grown in general in the past two decades. The upward trend in the publication of History of Medicine is shown from early 1900s articles in both databases. From the 1940s to the 1960s, the publication of articles fluctuated in PubMed and EMBASE. However, between 1970 and 1990, there was a moderate growth trend in publishing. Next, we have seen some gaps in the PubMed literature. The journal “Bulletin of the history of medicine” published the most articles in the field of the History of Medicine in both medical databases (Table 2). The publication of the journal Bulletin of the history of medicine was started in 1939 with quarterly frequency. They included the transactions of the annual meetings of the American Association of the History of Medicine.

**Table 2:** Top five journals with the most articles published in the field of the History of Medicine.

Journal titles in PubMed	*N*	Journal Titles in Embase	*N*
Société d'histoire de la pharmacie	499	Lancet	485
Lancet	492	British Medical Journal	465
British Medical Journal	475	Journal of the Royal Society of Medicine	370
Sovetskoe zdravookhranenie	442	Orvosi hetilap	356
Bulletin of the history of medicine	440	Bulletin of the history of medicine	355

The annual publication distribution is shown in Figure 2A. The average yearly publications calculated in excel were 668.23 ± 456.02 and 676.21 ± 480.44. In PubMed and Embase, the average annual growth rate is 1.54% and 0.34%, respectively. The literature growth rate has been positive or negative for the past years. This formula calculates the growth rate of annual publications distribution: Average Annual Growth Rate = (this year/last year)−1.

Figure 2B shows the sum of citations in the WoS and Scopus and the sum of the Altmetric Attention Score in the Altmetric Explorer during 1900–2021. Based on the data in the graphs, the Mean Collaboration Index shows the number of contributing authors in History of Medicine research, which best coverage of these two databases for medical history articles starts from the 1990s. Average citations per doc in WoS were 18.41 ± 93.56, Average citations per doc in Scopus were 8.81 ± 62.82, and Average Altmetric Attention Score (AAS) per doc in Altmetric Explorer was 17.68 ± 10.59.

**Figure 3:**
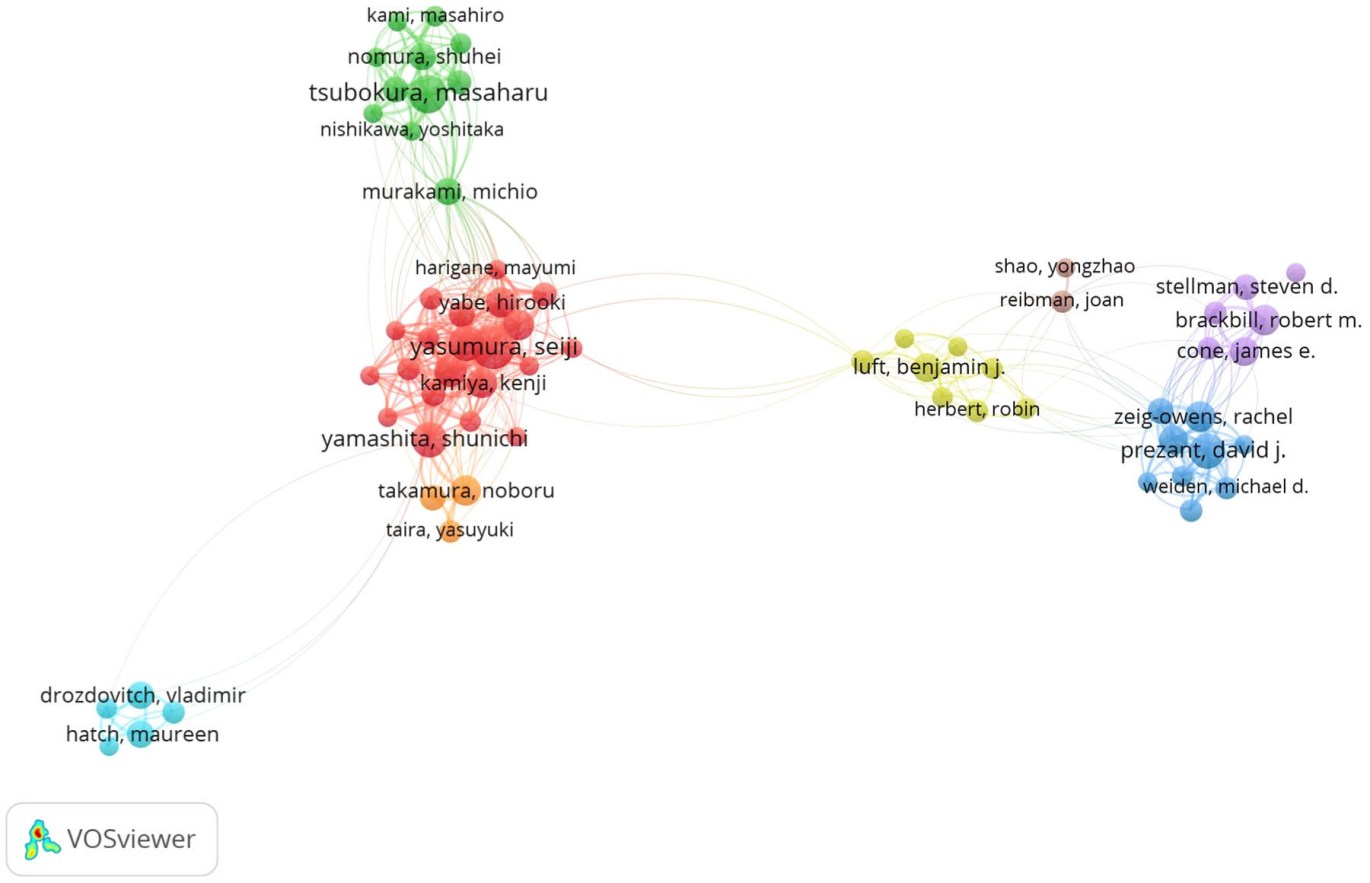
Visualization of the collaboration networks between researchers by History of Medicine in WoS database by VOSviewer.

Visualization of scientific collaboration researchers of History of Medicine in the WoS database showed 105,286 authors contributed. Out of the total 64,242 authors, only 90 authors contributed more than 20 times. Some of the 90 authors in the authorships network are not connected, and the most extensive set of related authors consists of 109 illustrated. Between the three normalization methods in VOSviewer software, the collaboration network of researchers is shown in eight clusters by the Fractionalization normalization method (Figure 3). The most prolific researcher, “Yasumura, Seiji” had 106 published studies. Respectively, “Tsubokura, Masaharu”, “Ohira, Tetsuya” and “Yamashita, Shunichi” are the most prolific writers by 84, 73, and 71 published studies. In network collaborations, all authors illustrated eight colored clusters (Figure 3).

These integrated data retrieved from the WoS citation databases showed that 16,282 documents in the History of Medicine field were written single‐authored. International co‐authorships in scholarly outputs were 10.89%. The coauthorship patterns showed the average coauthorship per document and the Collaboration Index calculated 2.64.

**Figure 4:**
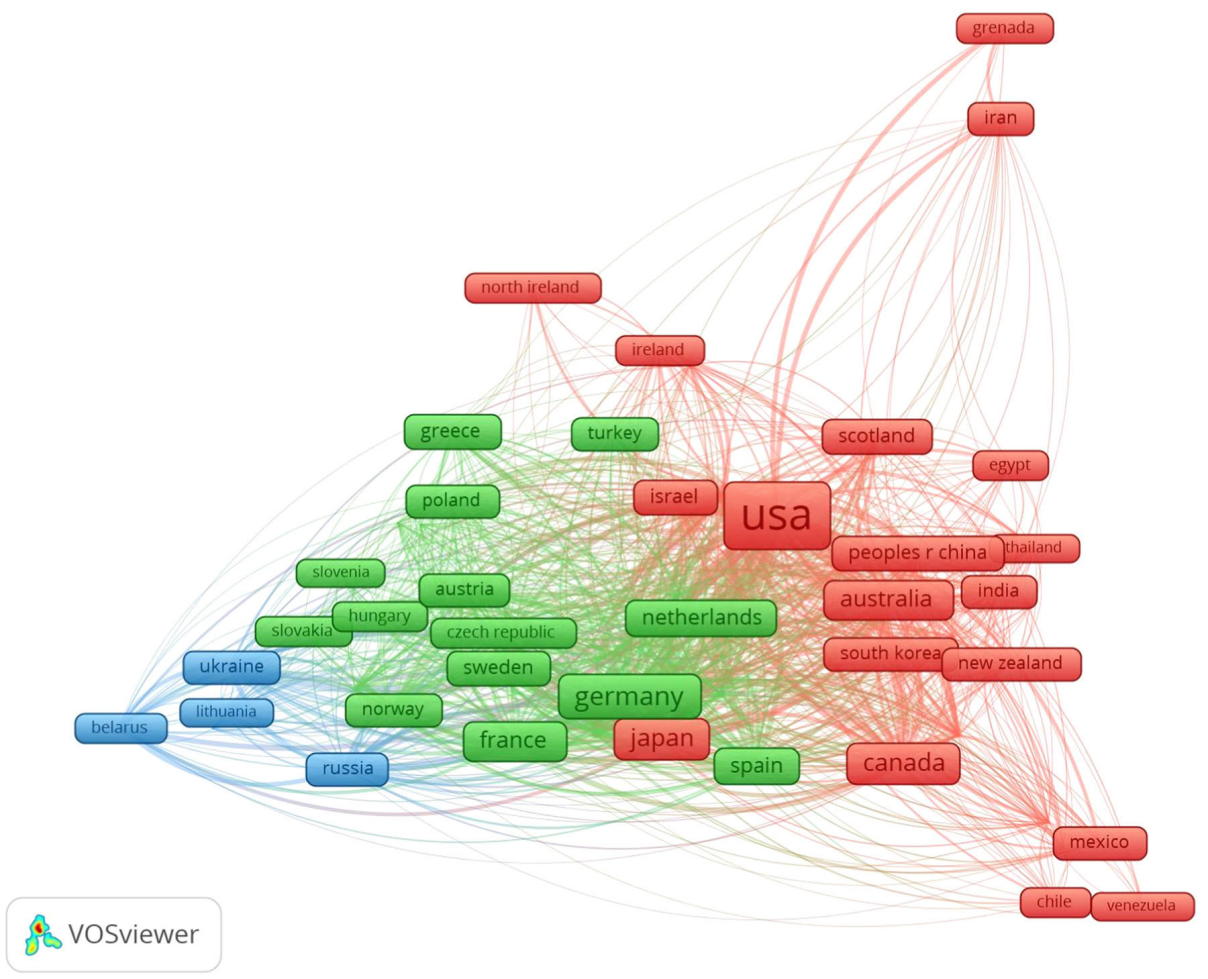
Visualization of the collaboration networks between countries in WoS database by VOSviewer.

Accordingly, History of Medicine researchers wrote scientific medical papers in collaboration with 157 countries including the United States, the United Kingdom, Germany, Japan, Canada, France and Italy, having more than thousands productive with History of Medicine research. Figure 4 illustrates the names of countries cooperating in History of Medicine research with 20 occurrences. The network collaborations of 58 countries clustered with six colors.

**Figure 5:**
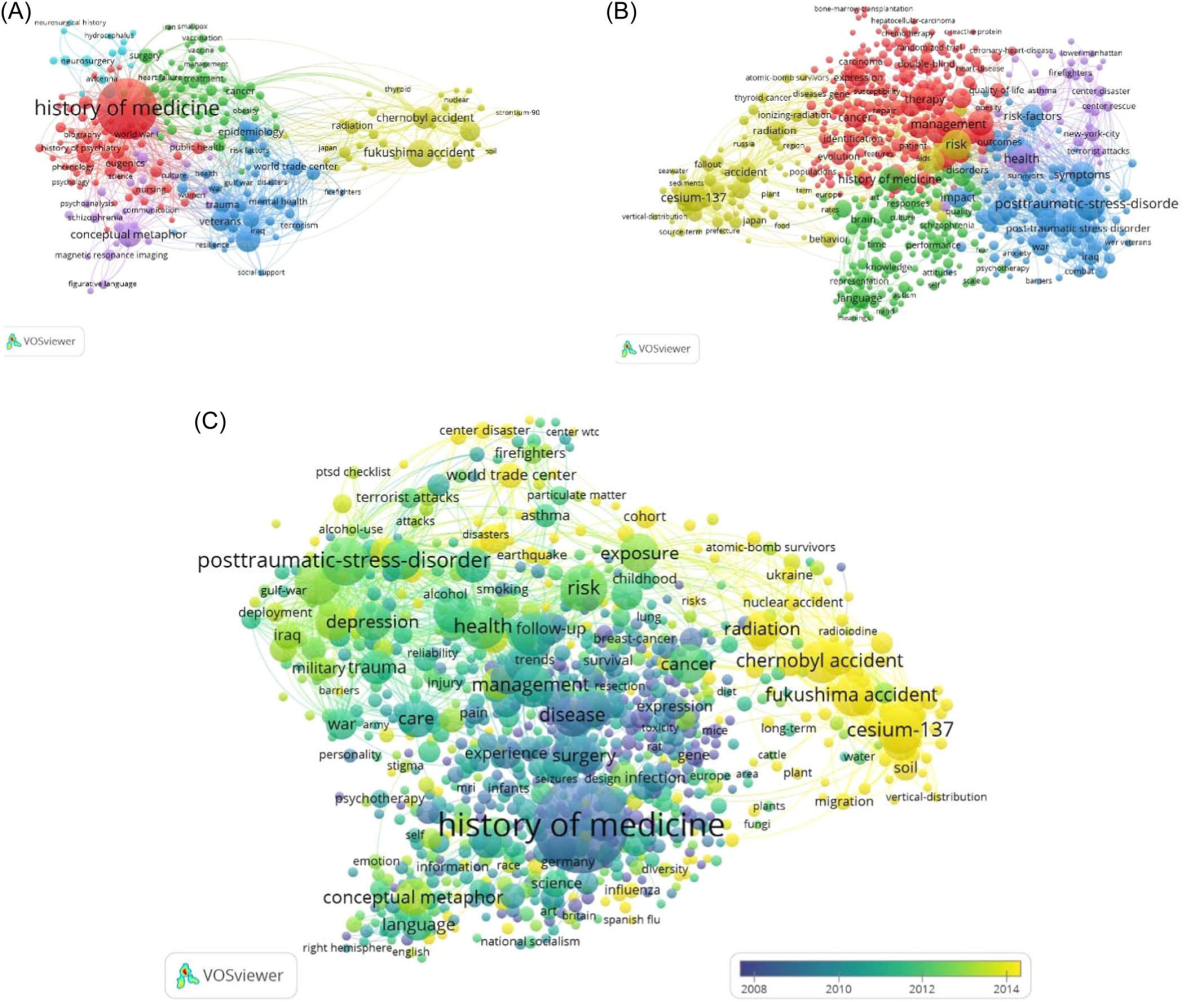
Visualization of the keywords in WoS database by VOSviewer. (A) Network visualization 241 Author keywords. (B) Network visualization 631 Keywords Plus. (C) Overlay visualization 903 all keywords.

The keywords analysis by co‐occurrence (Figure 5). The connection of items is determined based on the number of documents they occur together. The node size defines the number of published articles. The links between nodes represent relatedness. The color of a node represents the cluster it belongs to, and different colors represent different clusters.

Author keywords were 33,273, and 241 keywords have 20 occurrences visualized. Co‐occurrence illustrate based on author keywords showed seven clusters. The most frequent keywords around history of medicine included are metaphor, veterans, World Trade Center, Posttraumatic stress disorder (PTSD), and Chernobyl & Fukushima accident (Figure 5A).

Keywords Plus® is unique to Clarivate databases, and they are based upon a special algorithm for Web of Science. 627 of 32450 keywords plus were placed with 20 occurrences (Figure 5B).

Figure 5C shows unite analyses of all keywords (59995 keywords, 903 meet the threshold that the minimum number of occurrences of 20 keywords) that new concepts around History of Medicine research are yellow color: (1) disaster, (2) Chernobyl accident, (3) radiation, (4) mental‐health, (5) Soil.

### 3.2 Citation databases

After integrating the articles PubMed and Embase with EndNote software, the citation information was retrieved with their DOI and PMID fields. The new results of the Scopus database were 87501 articles in the timespan between 1908 until September 2022. Finally, records of citations were extracted from the Scopus database. These articles had received 39173 citations during this period. The highest number of citations was related to a published article entitled “New guidelines to evaluate the response to treatment in solid tumors”. Although the timespan of publication of articles in History of Medicine was between 1908 and 2022, more than 50 percent of the articles were published from 1990. Of the 87501 records of the Scopus database, there were 76.5% articles, 10.33% reviews, 3.75% editorials, 2.94% Note, 2.44% letters, 1.99% Conference Paper, and 1.89% Short Survey as document types.

### 3.3 Altmetric database

We only retrieved 19,954 articles from Altmetric Explorer. Analyzing the obtained data showed that the articles were presented on some social media. By attention breakdown, total mentions were divided into five categories: 303,336 mentions in social media, 16,228 mentions in other sources, 24,499 mentions in News and blogs, 7888 mentions in policymaking and patents, and 264 mentions in academic sources. Three thousand three hundred seventy‐four in the 16 social media records have no comments and an Altmetric Attention Score of zero; therefore, articles shared nobody at least once on the social media covered by the Altmetric Explorer database. The titles of other social media in the Altmetric Explorer had mentioned for the Medical History of Medicine articles, as follows in Table 3A.

**Table 3:** Descriptive analyses of the History of Medicine articles sharing in media (until 25 September 2022).

(A) Social media				
Social media	Total mentions	Range	Mean	Standard deviation
Twitter mentions	292,015	0–15,174	14.63	174.5
Facebook mentions	9042	0–156	0.78	3.00
Google+ mentions	1533	0–99	0.08	1.11
Reddit posts mentions	654	0–11	0.03	0.29
Sina Weibo mentions	16	0–4	0.00	0.04
Pinterest mentions	54	0–28	0.00	0.20
LinkedIn mentions	2	0–1	0.00	0.01
**(B) Other sources**				
Wikipedia mentions	15,508	0–238	0.78	2.93
Videos mentions	642	0–9	0.03	0.26
Q&A posts mentions	78	0–2	0.00	0.07
**(C) News and blogs**				
News mentions	19,669	0–239	0.99	6.70
Blogs mentions	4830	0–40	0.24	1.11
**(D) Policy and patents**				
Patents, mentions	4562	0–253	0.23	3.42
Policy documents mentions	3326	0–34	0.17	0.80
**(E) Academic sources**				
Faculty Opinions mentions	111	0–3	0.01	0.08
Peer reviews mentions	153	0–6	0.01	0.11

**Figure 6:**
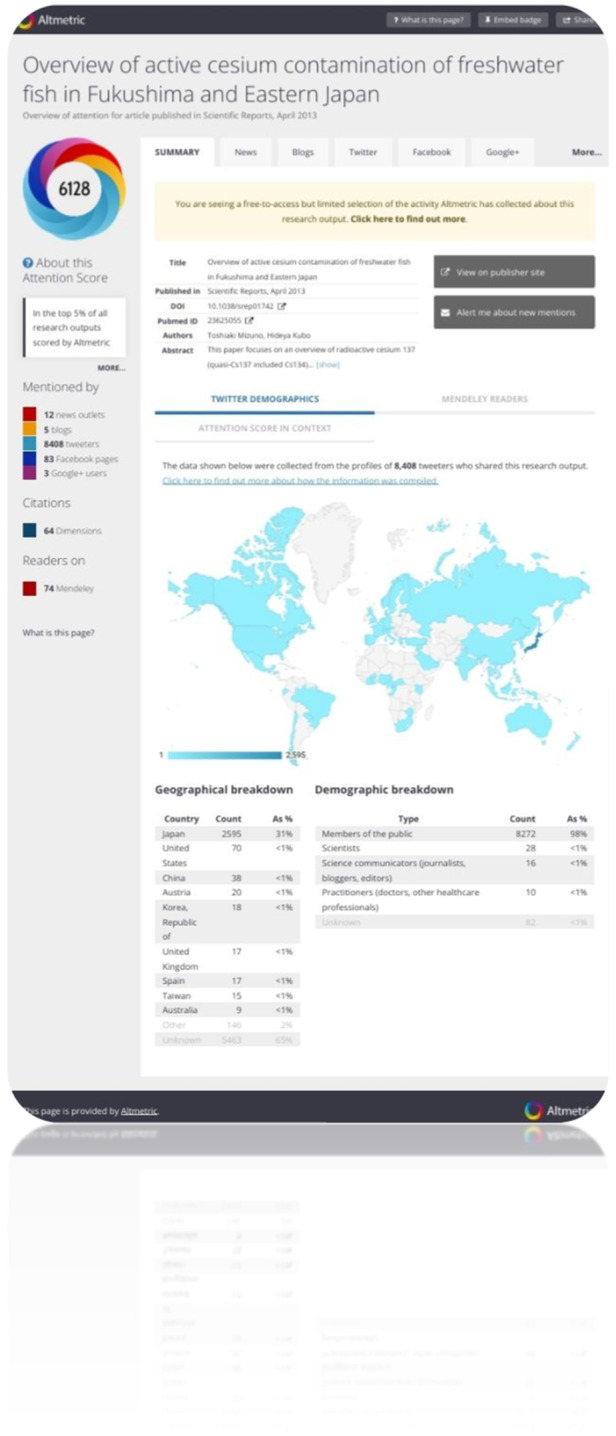
Screenshot of altmetrics webpage of the paper: “Overview of active cesium contamination of freshwater fish in Fukushima and Eastern Japan”.

The AAS is estimated with data from various social media sites. Also, the AAS is presented as a colorful donut where the colors represent the different sources of attention. Different colors of the donut obtain proportions of the respective sources, for example, blog posts are yellow, Twitter is light blue, and news sources are red. Suppose anybody mentioned findings published research in three blog posts, six Tweets, and two news outlets. The article receives an approximate Altmetric Attention Score of 37 (the score is weighted by source, 6 × 1+ × 5+2 × 8). Figure 6 points to a more qualitative analysis of the contexts in which the article's research has been mentioned (https://www.altmetric.com/details/1419082). In this figure, the 6128 score represents the highest AAS among the 19954 outputs included in this study. So, the donut illustrates the types (colors) in social media and the frequency (thickness of the stripes) of mentions on social media.

The highest amount of reflection was Twitter mentions, Dimensions citations, and Mendeley readers with 15,174, 13,966, and 6220 numbers, respectively. Table 3 shows the contribution of social media in sharing articles' descriptive and statistical analyses.

A Spearman's rho correlation coefficient was computed to assess the relationship between citation and the number of Altmetric mentions. The Altmetric Attention Score is to be associated with the rate of citation. The Spearman's rho for all papers was 0.057. Most correlations were statistically significant (*p* < 0.01), that means, there was a positive correlation between the variables (Table 4). All correlation coefficient between 0.017 and 0.289 were estimated “poor”, of course there is a correlation between citations and high all social media. A Spearman's rho correlation coefficient was computed to assess the relationship between citation and the number of Altmetric mentions. The Altmetric Attention Score is to be associated with the rate of citation. The Spearman's rho for all papers was 0.057 (Table 4).

**Table 4:** Statistical analyses of the History of Medicine articles by media impact and citations.

(A) Summary of the research impact with altmetric explorer databases and citation rate in scopus				
Social Media	Total mentions	Range	Mean	Standard Deviation
Altmetric attention score (AAS)	352757	0–6128	17.68	100.59
Mendeley readers	923223	0–6220	46.27	105.18
Dimensions citations	580014	0–13,966	29.07	125.72
Citation rate in Scopus	770937	0‐14,339	19.68	62.82

**(B) Correlations coefficient between citation rate and AAS (*N* articles** = **17,807)**			
Citation rate in scopus	Number of Mendeley readers	Number of dimensions citations	Altmetric attention score(AAS)
Correlation coefficient	0.482**	0.993**	0.057**
Sig. (two‐tailed)	0.000	0.000	0.000

** Correlation is significant at the 0.01 level (two‐tailed).

There is significant correlation between citations and social media, but all correlation coefficient between 0.017 and 0.289 were estimated “poor”. Of course, there is a correlation between citations and all social media into the Altmetric Explorer. Accordingly, most correlations were statistically significant (*p* < 0.01), that means, there was a positive correlation between the variables.

## 4 DISCUSSION

In this study, we attempted to illustrate the status of scientific research in the History of Medicine and network collaboration by data in the PubMed and EMBASE databases. The results of this study indicate that 107706 records during 1900‐2021 have been indexed in two medical databases, and the number of History of Medicine research has increased significantly in the last decades. Citation data of articles were re‐retrieved in WoS and Scopus. Respectively, the retrieved papers have an average of 18.41 and 8.81 citations per document; consequently, WoS articles received more citations than Scopus.

As well as the visualizations of authorship show who were involved in advancing the global science of the History of Medicine field and that these researchers can significantly impact this field's scientific progress as much as possible.^9^, ^13^ The mean Collaboration Index shows the number of contributing authors in History of Medicine research, which is less than the average number of co‐authors of all medical articles indexed in PubMed.^2^, ^14^, ^15^ Less than 10% of the research was conducted by a single author, indicating the interdisciplinary nature of History of Medicine research areas and increased scientific productivity with the scientific division of labor.^1^, ^15^, ^16^ Our results align with previous research that has investigated the connections between coauthorship and other factors, such as academic rank, departmental affiliation, citation count, and experience in the field.^16^, ^17^, ^18^, ^19^ Their results approved the relationship between the research teams and increased scientific output. As a result of previous studies, multi‐authorship and coauthorship are influential factors that increase the citation of articles.^2^, ^16^, ^20^ However, the scientific teamwork and coauthorship trends vary between groups, and one of the reasons may be due to differences in the scientific fields.

The results suggested that partnering with leading international institutions can enhance the research process by leveraging their expertize. The United States, the United Kingdom, and Germany have been the dominant contributors to the field of History of Medicine in terms quantity. However, advancements in scientific and social networks now enable greater global interactions across a wider geographic scope, removing limitations imposed by distance.^3^, ^21^

Currently, there is a growing desire among social media users for “information,” encompassing various aspects of life, including academic endeavors.^4^, ^22^ The various features available on social media platforms enable users to swiftly comment, share scientific articles, and engage in societal discussions. According to the research, Twitter received the highest level of attention among social media platforms in this particular field. This aligns with previous studies that have also found Twitter to be the most prominent platform in terms of user engagement,^13^, ^23^, ^24^ However, Twitter, launched in 2006 as a social media, is the most studied metric in the Altmetrics literature.^5^ Today, Altmetric Explorer coverage of articles on Twitter was about 40% in 2018. The finding of this study showed very poor correlation that resulted in mentions causing the citations (0.04). Also, a meta‐analysis review of studies have shown there is a weak correlation between citation rate of articles and introduction in social media.^25^

The findings indicated that News and Wikipedia were ranked second and third, respectively, in terms of spreading research on the History of Medicine. There was a correlation between the rate of article citations and mentions in News (*p* = 0.217) and Wikipedia (*p* = 0.132). Previous scientific studies have demonstrated the beneficial impact of social media in facilitating knowledge sharing, enhancing education, and showcasing innovative treatment approaches, particularly by highlighting current scientific research through citations.^26^

The findings of this study indicated that there is limited diversity and insufficient attention given by researchers in the field of medical history on social media platforms. For example, in articles published in the field of History of Medicine, the 17% social media have a mean Altmetric Attention Score of zero. Hence, Altmetrics employing a vibrant circular graph not only assess the societal influence of a publication but also motivate and endorse its achievement. Visually, the vivid colors and scores are highly noticeable, capturing the attention of scientists and other pertinent individuals involved.

The field of History of Medicine is highly important and receives significant attention for its research output. Social media usage is widespread among the health community, as evidenced by various metrics such as Dimensions citations, Mendeley readers, and AAS. The number of scientific citations a research article receives contributes to its attractiveness and visibility on social media, while analyzing comments and mentions can provide insights into its perceived quality. This study also found a positive and significant correlation between citations and Altmetric Attention Score. However, it's important to note that the relationship between citations and these variables is bidirectional, meaning that an increase in one platform leads to an increase in the other. Future studies could explore the demographic characteristics of social media users for further analysis. The findings of this research have implications for decision‐making, research evaluations, and policy formulation, providing incentives and guidance. Social networks facilitate direct and rapid communication between Historians of Medicine researchers and various stakeholders such as physicians, physical therapists, practices and hospitals, scientific societies, scientific journals, insurance companies, medical technology and pharmaceutical companies, and patients.^6^, ^21^, ^27^, ^28^, ^29^ Identifying gaps in research on the history of medicine is crucial for gaining a comprehensive understanding of the field's trends and changes over an extended period. Research gaps refer to areas within the medical domain that have significant potential for further investigation but have not been explored by researchers specializing in medical history. The number of published medical articles has seen a notable increase between 1990 and 2021, driven by growing interest among academics in historical aspects of medicine. This upward trend is expected to continue, leading to a greater volume of scientific studies in the coming years. By examining the status of research areas and fostering collaboration, new perspectives on scientific communication behaviors can be uncovered. Sharing research articles through social scientific networks can enhance the visibility of works in the history of medicine research, which significantly influences article citations. Social networks often serve as platforms for direct and rapid communication exchanges between historians of medicine, physicians, physical therapists, medical practices and hospitals, scientific societies, scientific journals, insurance companies, medical technology and pharmaceutical companies, as well as patients.

## 5 CONCLUSION

This study aims to provide insights into the history of medicine research output worldwide and its impact on the field. The research identifies influential articles, authors, and countries, and analyzes the topics and trends in history of medicine research using bibliometric and altmetric approaches. Collaboration among active medical authors is emphasized as a key factor in advancing the field. The study also examines the status of research areas and collaboration, revealing new perspectives on scientific communication behaviors and informing policy‐making and strategic research management. Sharing research findings on social scientific networks, such as twitter and Mendeley, increases the visibility of historical medical works and influences article citation. The findings can guide decision‐making, research evaluation, and the formulation of research policies. The study emphasizes the importance of continuous efforts to strengthen the output and impact of history of medicine publications, while upholding ethical research practices and promoting transparency and integrity in scientific research.

## AUTHOR CONTRIBUTIONS

**Jamal Rezaei Orimi**: Conceptualization; investigation; methodology; project administration; supervision; writing—original draft; writing—review and editing. **Mohammad Hossein Asadi**: Conceptualization; methodology; validation; writing—original draft. **Forouhe Jafari**: Conceptualization; investigation; methodology; resources; writing—original draft. **Aboozar Ramezani**: Conceptualization; data curation; formal analysis; investigation; methodology; project administration; resources; software; supervision; validation; visualization; writing—original draft. **Seyyed Amir Hossein Latifi**: Conceptualization; methodology; resources; writing—original draft. **Azam khosravi**: Conceptualization; methodology; resources; writing—original draft. **Seyed Abdollah Mahmoodi**: Data curation; methodology; project administration; resources; writing—original draft. **Mehdi Salehi**: Conceptualization; methodology; project administration; resources; writing—original draft. **Hasan Siamian**: Conceptualization; data curation; formal analysis; investigation; methodology; project administration; resources; software; supervision; validation; visualization; writing—original draft; writing—review and editing.

## CONFLICT OF INTEREST STATEMENT

The authors declare no conflict of interest.

## ETHICS STATEMENT

The ethics committee of Arak University of Medical Sciences approved the study under the registration number IR. ARAKMU. REC.1401.152.

## TRANSPARENCY STATEMENT

The lead author Hasan Siamian affirms that this manuscript is an honest, accurate, and transparent account of the study being reported; that no important aspects of the study have been omitted; and that any discrepancies from the study as planned (and, if relevant, registered) have been explained.

## Data Availability

All the data used in this study are present within the study itself. Neanwhile, Data will be available on reasonable request to the corresponding author.

## ENDNOTE

* Twitter, Facebook, Google+, Reddit posts, Sina Weibo, Pinterest, LinkedIn, Wikipedia, Videos, Q&A posts, News, Blogs, Patents, Policy documents, Faculty Opinions, Peer reviews, Syllabi, Number of Mendeley readers, Number of Dimensions citations. https://www.altmetric.com/about-altmetrics/what-are-altmetrics.
